# Chromatin-Associated Protein Sugp2 Involved in mRNA Alternative Splicing During Mouse Spermatogenesis

**DOI:** 10.3389/fvets.2021.754021

**Published:** 2021-10-18

**Authors:** Junfeng Zhan, Jianbo Li, Yuerong Wu, Panfeng Wu, Ziqi Yu, Peng Cui, Mofan Zhou, Yumin Xu, Tingyu Jin, Ziye Du, Mengcheng Luo, Cong Liu

**Affiliations:** ^1^Department of Urology, Zhongnan Hospital of Wuhan University, Wuhan, China; ^2^Department of Tissue and Embryology, School of Basic Medical Sciences, Wuhan University, Wuhan, China; ^3^Hubei Provincial Key Laboratory of Developmentally Originated Disease, Wuhan, China; ^4^Center for Animal Experiment, Wuhan University, Wuhan, China

**Keywords:** spermatogenesis, chromatin-associated protein, SUGP2, alternative splicing, male germ cell

## Abstract

Mammalian spermatogenesis is a highly ordered process that is determined by chromatin-associated moderators which still remain poorly understood. Through a multi-control group proteomics strategy, we confirmed that Sugp2 was a chromatin-associated candidate protein, and its signal arose along spermatogenesis. The expression results showed that Sugp2, which is mainly expressed in the testis, had two transcripts, encoding one protein. During spermatogenesis, Sugp2 was enriched in the nucleus of male germ cells. With the depletion of Sugp2 by CRISPER-Cas9 technology, we found that Sugp2 controlled a network of genes on metal ion and ATP binding, suggesting that alternative splicing regulation by Sugp2 is involved in cellular ion and energy metabolism during spermatogenesis, while it had a little effect on meiotic progression and male fertility. Collectively, these data demonstrated that, as a chromatin-associated protein, Sugp2 mediated the alternative splicing regulatory network during spermatogenesis.

## Introduction

Spermiogenesis is recognized as one of the most complex biological processes. This process involves two main elements: (1) the cellular extrinsic stimuli, including organic or inorganic nutrients and growth factors produced in a supportive niche, and (2) intrinsic factors, mainly related to cellular metabolism regulated by gene expression. Alternative pre-mRNA splicing (AS) is critical for gene expression, and extensive AS variants are enriched in different developmental stages of mouse spermatogenesis. This post-transcriptional regulation promotes the establishment of a regulatory mechanism for changes in both internal and external environments of the cell during spermatogenesis and also promotes male gamete production (1–4).

As an indispensable component of gamete production, meiosis is a specialized type of cell division that creates haploid cells from diploid progenitors and ensures genetic diversity through homologous recombination (5). During meiosis, recombination is initiated by programmed DNA double-strand breaks (DSBs) generated by Spo11 (6) and TOPOVIBL (7). Subsequently, meiotic DNA DSBs will cause the phosphorylation of histone variant H2ax (γH2ax) at the Ser-139 residue, which is an early cellular response to the induction (8), recruit a series of recombination proteins to form recombination foci, and promote homologous chromosome synapsis during the zygotene stage (9, 10). Strand invasion of the 3′ single-stranded DNA, shielded by RPA protein complex, into the duplex of the homolog is mediated by recombinase Dmc1 and Rad51 (10–12). At the pachytene stage when the homologous chromosomes are fully synapsed, recombination foci continue to mature and are eventually resolved into crossover or non-crossover events, which ensure the correct separation of homologous chromosomes (5).

The biochemical mechanism of meiosis is inseparably interconnected with a chromatin structure. Most of the regulatory factors can integrate into the chromatin body and act by various mechanisms (13). Our method for the isolation of spermatogenetic chromatin-associated proteins had identified many important male reproductive factors, such as Meiob, Scml2, and Sdx, involved in meiotic recombination, histone modification, and gene transcription, respectively (14–16). Since spermatogenesis is a multicellular and multi-stage process, the regulation mechanisms, especially in special cell types or at special stages, are still largely unknown.

In this study, adapting with proteomic data on spermatogenetic chromatin-associated proteins, we focused on Sugp2 which may be involved in RNA regulation. We systematically analyzed the expression and localization of Sugp2 in mouse testes by generating Sugp2 antibody and explored its function during spermatogenesis through a Sugp2-deficient mouse model based on CRISPER-Cas9 technology. This research would help us better understand the regulation mechanism in spermatogenesis.

## Materials and Methods

Unless otherwises specified, the drugs used in the experiments are from Sigma Aldrich (United States).

### Animals

All mice were housed in the pathogen-free animal facility of Wuhan University. All animal studies were reviewed and approved by the Institutional Animal Care and Use Committee of Wuhan University. The *Sugp2* knockout mice were purchased from Jiangsu GemPharmatech Company in China. The mouse genotype was identified by extracting DNA from mouse tail and combining with PCR. The primers used for genotyping are listed in Table 1. All the animal experiments were performed under the animal ethical guidelines of the Institutional Care and Wuhan University.

**Table 1:** Primers used in this research.

**Name**	**Primer**
GAPDH	F: CCCCAATGTGTCCGTCGTG
	R: TGCCTGCTTCACCACCTTCT
Sugp2-Transcript1	F: AAACCCAAGGACATGGAGTTT
	R: CTATTTGGCCCGCTTGT
Sugp2-Transcript2	F: AAACCCAAGGACATGGAGTTT
	R: AGGGCTGGGGCTAGTAAGCTA
Sugp2 identification	F: CACAGCGGGTAGCAGGTAAG
	R-WT: GTGGAGCATGACAAATGGTCACATA
	R-KO: CTGCCCTTATCTATGACGCTATGG
Sugp2-antibody-F	aaatcggatggttcaactagtGAGCCTGCCAAACCCTGTCC
Sugp2-antibody-R	gtggtggtggtggtgctcgagCATGCGCTGGCGGAAGACGT

### Purification and Screening of Chromatin-Associated Proteins

The purification of chromatin-associated proteins was carried out as (15) with modifications. One hundred milligrams of testis tissues from different groups was homogenized in 1 ml of lysis buffer 1 (250 mM sucrose, 10 mM Tris-HCl, pH 8.0, 10 mM MgCl_2_, 1 mM EGTA, and 1X protease inhibitor cocktail) on ice. After centrifugation at 300 *g* for 5 min, the supernatant containing cytoplasmic components can be collected, and the pellets were homogenized in buffer 2 (buffer 1 plus 1% Triton X-100) and centrifuged at 100 *g* to remove the nuclear membrane. The supernatant that contains chromatin-associated proteins was layered over a 1.7-M sucrose cushion and centrifuged at 50,000 *g* for 1 h in a Beckman SW32Ti rotor. The resulting pellets were further purified by resuspending in Tris buffer C (10 mM Tris HCl, 0.2 mM EDTA, 0.1% Triton X-100, and 1X protease inhibitor cocktail) and centrifugation at 1,300 *g*, and the precipitate was used for screening.

The purified chromatin-associated proteins were shipped in solidified carbon dioxide and subjected to high-performance liquid chromatography followed by HRMS at the National Facility for Protein Science in Shanghai, Zhangjiang Lab, China. The proteomics analysis from different testes samples was conducted by the PEAKS software (17).

### Western Blot Analysis

Tissues were lysed in RIPA buffer (Beyotime Biotechnology) with 1X protease inhibitor cocktail (Roche) on ice. After centrifuging the lysate at 12,000 *g* for 20 min, the supernatant was added with 5× SDS loading buffer (Beyotime Biotechnology) and allowed to boil for 10 min to get the protein samples. Then, the proteins in the samples were separated by SDS-PAGE and transferred onto polyvinylidene difluoride membranes. The membranes were blocked for 1–2 h in 5% skimmed milk in TBST (TBS, 0.1% Tween-20) and incubated with primary antibodies overnight at 4°C. After three times of washing in TBST, the membranes were incubated with HRP-conjugated secondary antibodies anti-mouse or anti-rabbit for 1 h at RT. The signals were developed with SuperSignal™ West Pico PLUS Chemiluminescent Substrate (Thermo Fisher Scientific) and captured with Amersham Imager 600 (GE Healthcare Life Sciences). Information on the antibodies used are shown in Table 2.

**Table 2:** The antibody dilution ranges used in present research.

**Protein**	**Producer**	**Application**	**Dilution**
Sug2	Our Lab	Western blot	1:100
		Immunofluorescence	1:50
Sycp3	Our Lab	Western blot	1:100
		Immunofluorescence	1:50
Sycp1	Our Lab	Western blot	1:100
		Immunofluorescence	1:50
Hspa5	Abclonal	Western blot	1:100
Vdac1	Abclonal	Western blot	1:100
Ddx4	Abcam	Immunofluorescence	1:100
Plzf	Millipore	Immunofluorescence	1:50
γ-H2ax	Abclonal	Immunofluorescence	1:100
Rad51	Our Lab	Immunofluorescence	1:50
Rpa1	Our Lab	Immunofluorescence	1:100
Mlh3	Our Lab	Immunofluorescence	1:50
Gapdh	Proteintech	Western blot	1:20,000
CoraLite-488 conjugated Affinipure Goat Anti-Mouse secondary antibody	Proteintech	Immunofluorescence	1:200
CoraLite-594 conjugated Affinipure Goat Anti-Mouse secondary antibody	Proteintech	Immunofluorescence	1:200
HRP-conjugated Affinipure Goat Anti-Mouse IgG(H+L)	Proteintech	Western blot	1:10,000
HRP-conjugated Affinipure Goat Anti-Rabbit IgG(H+L)	Proteintech	Western blot	1:10,000

### Phylogenetic Analysis

The sequences of Sugp2 from different species were downloaded from the NCBI database, and these sequences were used to construct a phylogenetic tree using the Molecular Evolutionary Genetic Analysis (MEGA X) software (18).

### Generation of Sugp2 Polyclonal Antibodies

The sequence encoding aa 723–1,056 of mouse Sugp2 was amplified by PCR and ligated into the Pet42b plasmid by homologous recombination. The reconstructed Pet42b plasmid was transformed into the BL3 strain, and the GST-fused Sugp2 expression was induced by isopropylthio-β-galactoside. After the bacterial cells were lysed by sonication, the affinity of Sugp2 antigen was purified with Ni beads and purified with dialysis. Finally, Sugp2 antibody were obtained by immunizing rabbits.

### Immunofluorescence, Histological, and Surface Nuclear Spread Analyses

For immunofluorescence analysis, testes were fixed in Bouin's solution at room temperature overnight, dehydrated in 30% sucrose (in 1X phosphate-buffered saline) overnight, embedded with NEG-50 (Thermo Fisher Scientific) at −80°C, and sectioned into 8-μm cryosections. After antigen-retrieval in 0.1 M sodium citrate buffer (pH 6.0), the sections were permeated in TBS (20 mM Tris, pH 7.4, 150 mM NaCl) with 1%Triton X-100 for 30 min at room temperature. Then, the sections were blocked in TBS containing 10% goat serum (Solarbio Life Sciences) and a subsequent first antibody incubation overnight. After three times of washing in TBS, the sections were incubated with CoraLite 488- or 594-conjugated Affinipure Goat Anti-mouse or Anti-rabbit secondary antibodies (Proteintech). The samples were then washed three times with TBS, counterstained with DAPI (Solarbio Life Sciences), and covered with coverslips.

For histological analysis, the fixed testes were processed with a series of dehydration, embedded with paraffin, and sectioned at 5–8 μm. The sections were stained with hematoxylin and eosin.

The surface nuclear spread adopted the method previously reported (19). Briefly, testicular tubules were extracted in a hypotonic treatment buffer (30 mM tris, 50 mM sucrose, 17 mM trisodium citrate dihydrate, 5 mM EDTA, 0.5 mM dithiothreitol, and 1 mM phenylmethylsulfonyl fluoride). The isolated cells were re-suspended in 0.1 M sucrose and then spread on a glass slide with a thin layer of paraformaldehyde solution containing Triton X-100. Then, the slides were stained by the immunofluorescence staining method described earlier. The dilution ranges of the different antibodies used in the present study are listed in Table 2. Histological and fluorescence images were captured with Axio Imager 2 Microscope (Zeiss).

### RNA Sequencing

The testes were isolated, and 1 μg RNA per sample was used for library preparation. Sequencing libraries were generated using NEBNext^®^ UltraTM RNA Library Prep Kit for Illumina^®^ (New England Biolabs) following the recommendations of the manufacturer. Briefly, mRNA was purified from total RNA using magnetic beads. Fragmentation was carried out using divalent cations under elevated temperature. First-strand cDNA was synthesized using random hexamer primer and M-MuLV Reverse Transcriptase (RNase H-). A second-strand cDNA synthesis was subsequently performed using DNA Polymerase I and RNase H. The remaining overhangs were converted into blunt ends *via* exonuclease/polymerase activities. After adenylation of the 3′ ends of DNA fragments, NEBNext Adaptor with a hairpin loop structure was ligated to prepare for hybridization. The library fragments were purified with AMPure XP system (Beckman Coulter), size selected and ligated with an adaptor using 3 μl USER Enzyme (New England Biolabs) at 37°C for 15 min followed by 5 min at 95°C before the PCR. The PCR was performed with Phusion High-Fidelity DNA polymerase, Universal PCR primers, and Index (X) Primer. Finally, the PCR products were purified with AMPure XP system. After cluster generation, the library preparations were sequenced on an Illumina Novaseq platform, and 150-bp paired-end reads were generated. All RNA sequencing data have been deposited at SRA, and the BioProject ID/accession no. is PRJNA752112.

### Bioinformatic Data Analysis

Raw data (raw reads) of fastq format from WT and Sugp2 KO were firstly processed through in-house perl scripts. In this step, clean data (clean reads) were obtained by removing reads containing adapter, reads containing ploy-N, and reads with low quality from raw data. All the downstream analyses were based on the clean data with high quality. Using Hisat2 v2.0.5 (20), an index of the reference genome was built, and paired-end clean reads were aligned to the reference genome. The mapped reads of each sample were assembled by StringTie v2.1.3 (21) in a reference-based approach and guided by transcriptome annotation (GRCm38, GENCODE release M25). Taking the calculated transcripts per million (TPM) as the baseline for difference expression mapping, genes with TPM < 1 were excluded. The analysis of differential expression used the log2 of the calculated TPM fold change between WT and Sugp2 KO.

### Statistics

Data was analyzed with one-way ANOVA followed by a Student–Newman–Keuls comparison test using SPSS 20.0 software (SPSS Inc.). Significance was set at *P* < 0.05 for all tests.

## Results

### Sugp2 Was a Putative Spermatogenetic Chromatin-Associated Protein

**Figure 1:**
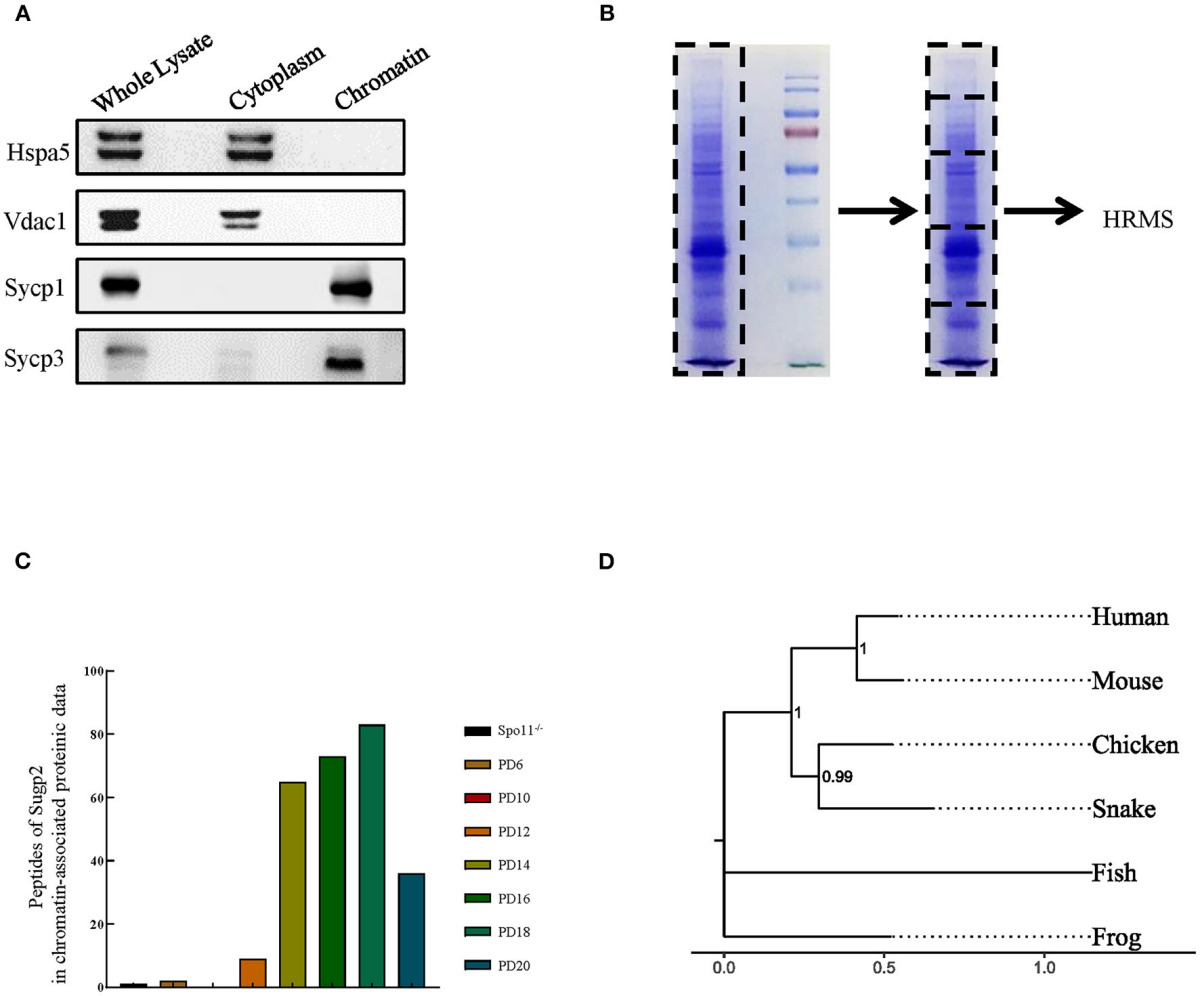
Sugp2 was a putative spermatogenetic chromatin-associated protein by proteomic identification. **(A)** Purity assessment of isolated testis chromatin-associated proteins by western blot. Sycp1 and Sycp3 are components of meiotic synapsis complex and associated with chromatin. Hspa5 and Vdac1 are cytoplasmic protein controls. **(B)** Identification of chromatin-associated proteins by high-resolution mass spectrum. **(C)** Peptide counts of Sugp2 in testis from Spo11 knockout (Spo11^−/−^) and different postnatal groups. **(D)** Phylogenetic tree for Sugp2 protein estimated by neighbor joining method. The tree was constructed by MEGA. Each name at the terminus represents the species from which the protein originated.

Using our previously reported method for meiotic chromatin-associated protein extraction, we obtained high-purity chromatin-related proteins (Figure 1A). Sycp1/3, as synaptonemal complex (SC) proteins associated with meiotic chromatin, was present in the chromatin fraction, whereas Hspa5 and Vdac1 were in cytoplasmic components as marker proteins for the endoplasmic reticulum and the mitochondria, respectively. After extraction, the chromatin-associated proteins were separated by SDS–polyacrylamide gel electrophoresis (PAGE) and analyzed by high-resolution mass spectrometry (HRMS; Figure 1B). In the present study, we analyzed eight sets of data. Spo11^−/−^ testis was used to screen for proteins involved in meiotic DNA damage repair processes since Spo11 knockout induced spermatocytes that lacked meiosis-specific DSBs and subsequent DNA damage repair processes and were arrested in the zygotene-like phase. PD6 mouse testis contains only spermatogonia but no spermatocytes, which can be used to screen meiotic spermatocyte-related proteins. While PD10, 12, 14, 16, 18, and 20 mouse testes contain spermatocytes at different developmental stages during the first wave of spermatogenesis, they can be used to screen proteins at different meiotic stages.

By analyzing and comparing the proteomic data, we identified one putative spermatogenetic chromatin-associated protein—Sugp2, which showed high peptide abundances from PD12 to PD20 proteomic data but had a low level in Spo11^−/−^ and PD10 groups (Figure 1C). Among different species, Sugp2 had high protein sequence conservativeness, especially between human and mice (Figure 1D), implying the conservation of its biological function.

### Sugp2 Was Primarily Expressed in Mouse Testis

**Figure 2:**
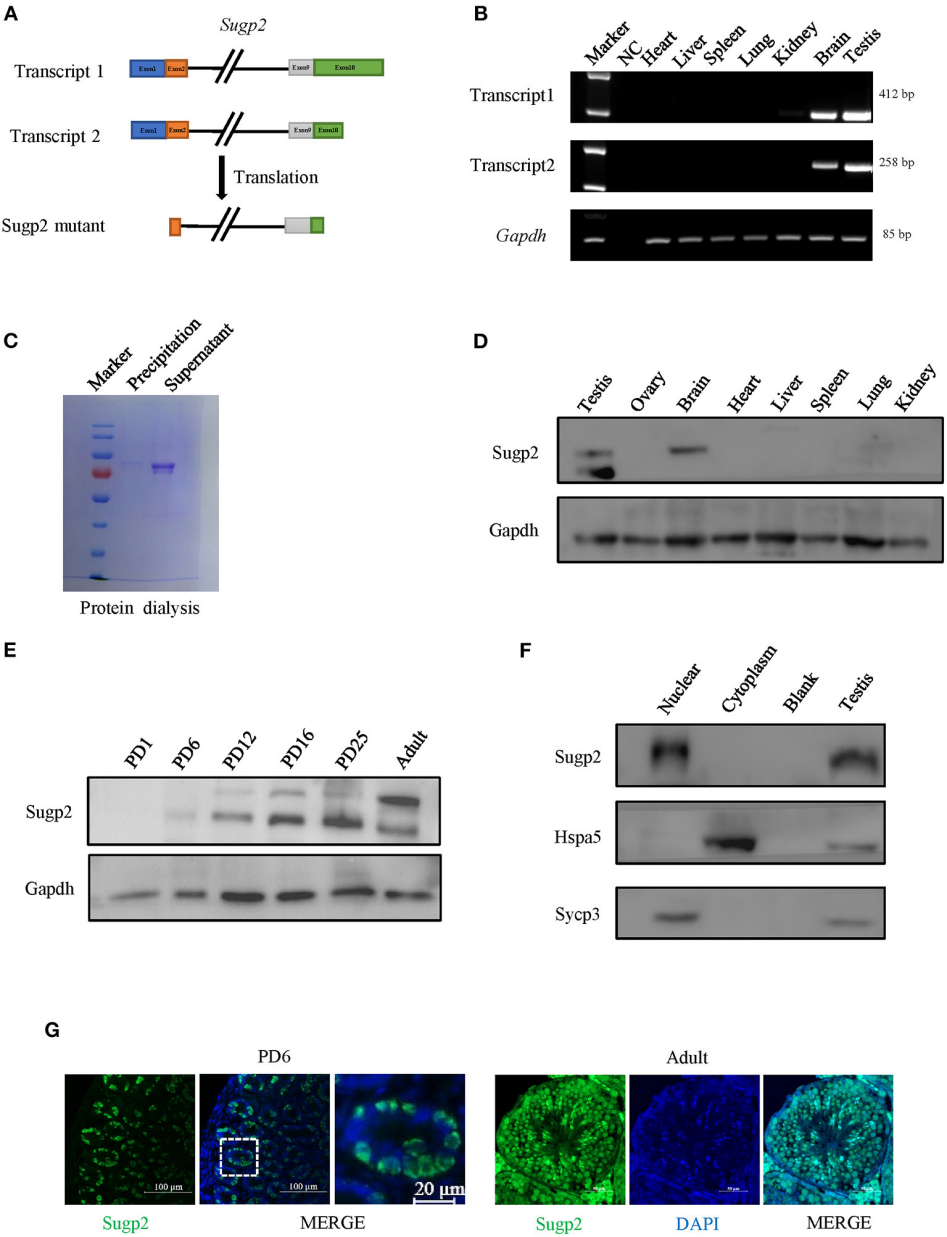
Expression of Sugp2 during mouse spermatogenesis. **(A)** Transcription and translation information of Sugp2. **(B)** Identification of Sugp2 isoforms by RT-PCR. **(C)** Purification of Sugp2 antigen by dialysis. The expression pattern of Sugp2 in different tissues **(D)** and testes at different stages **(E)**. The expression of Sugp2 in mouse testis was confirmed by western blot **(F)** and immunofluorescence **(G)**.

Proteomic data showed that Sugp2 was highly expressed in the process of meiosis, suggesting that it may play a role in meiosis. To obtain more information on Sugp2, we first verified the expression of Sugp2 in various tissues at expression and translation levels. There are two different transcripts of *Sugp2* gene according to National Center of Biotechnology (NCBI), which vary at the 5′ end but encode the same protein (Figure 2A). Through RT-PCR detection, both transcripts of Sugp2 existed and were primarily transcribed in adult testis and brain (Figure 2B). Due to the lack of effective commercial antibodies to mouse Sugp2, we made a rabbit polyclonal antibody with good effect against it by immunization with purified Sugp2-truncated protein (Figure 2C). Western blot analysis revealed that Sugp2 was exclusively expressed in testis and brain, and its expression increased along with the progression of the first wave of spermatogenesis (Figures 2D,E). What is more, the nuclear and cytoplasmic separation assay results further confirmed that Sugp2 co-localized with chromatin of the testis (Figure 2F). At postnatal day 6 (PD6), Sugp2 was enriched in certain cells located in the basement membrane of the seminiferous tubules, while in adult testis Sugp2 was also expressed in the spermatogenic cells located in the middle of the seminiferous tubules (Figure 2G).

### Sugp2 Was Enriched in the Nucleus of Male Germ Cells

**Figure 3:**
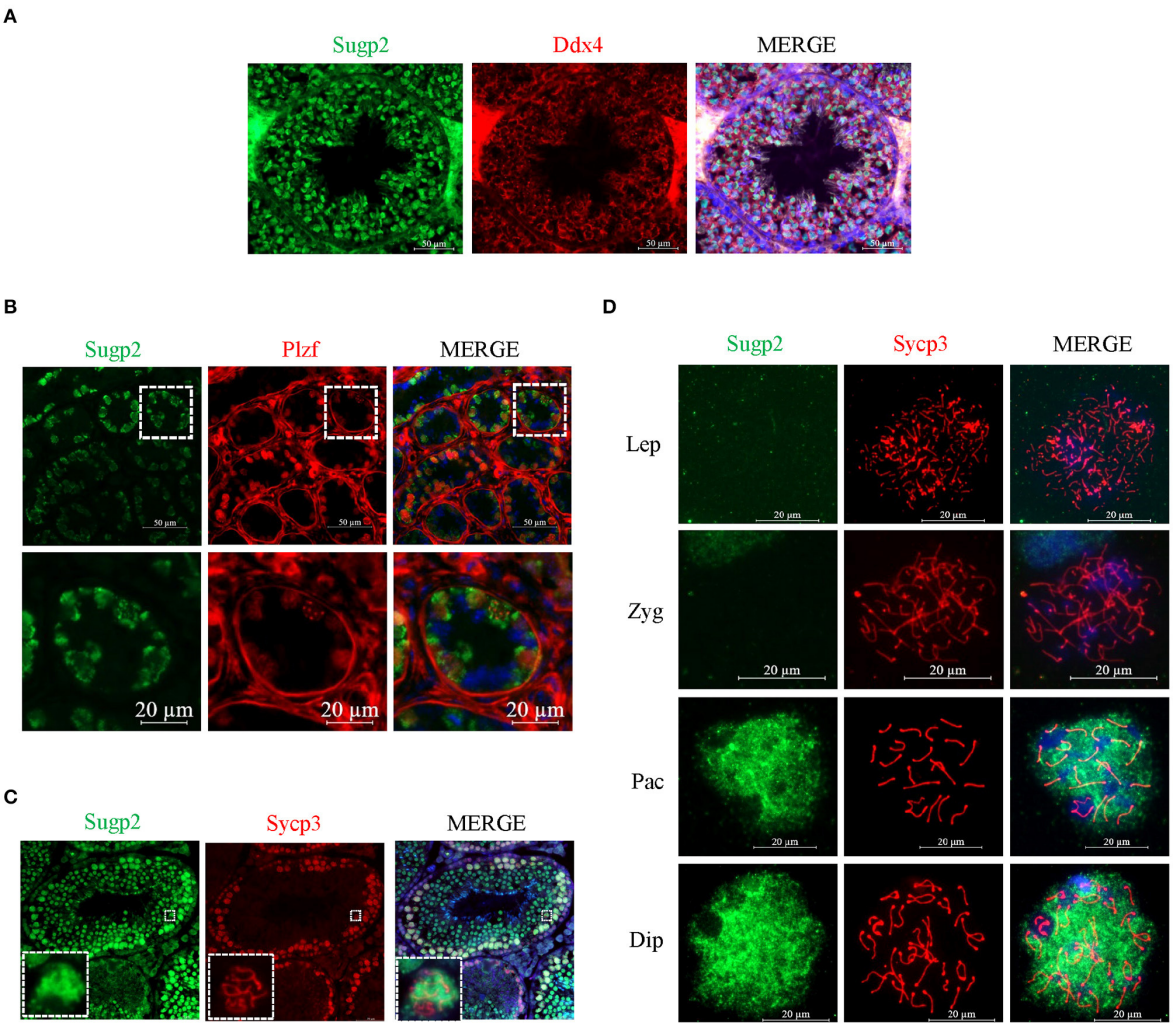
Localization of Sugp2 in mouse testis. Frozen sections of adult **(A,C)** and PD6 **(B)** testes were co-stained with rabbit anti-Sugp2 and mouse anti-Ddx4 **(A)**, Plzf **(B)**, or Sycp3 **(C)** antibodies. Ddx4, Plzf, and Sycp3 were used as germ cells, spermatogonia, and spermatocyte markers, respectively. The DNA was stained with DAPI. Scale bar = 20 μm. **(D)** Distribution pattern of Sugp2 on the chromatin of spermatocytes from leptotene through diplotene stages.

To further clarify the localization of Sugp2 in mouse testes during spermatogenesis, we performed double-staining with Ddx4, Plzf, and Sycp3, which indicates total germ cell, spermatogonia, and spermatocytes, respectively. By co-staining with Ddx4, we could see the colocalization of Sugp2 and Ddx4, indicating that Sugp2 was mainly expressed in male germ cells and located in the nucleus (Figure 3A). Furthermore, the co-localization of Sugp2 with Plzf and Sycp3 indicated that Sugp2 was highly expressed in germ cells at different levels of maturity, including spermatogonia, spermatocytes, and haploid sperm cells. Interestingly, when co-stained with Sycp3, the Sugp2 signal was not uniform, with some blanks in certain areas (Figures 3B,C). In addition, immunostaining of spread spermatocyte nuclei showed diffuse Sugp2 immunofluorescent signal from the pachytene to diplotene stages, while the signal was mainly located in the autosome area but was excluded from the region of XY body (Figure 3D).

### The Deficiency of Sugp2 Has a Little Effect on Male Fertility and Spermiogenesis

**Figure 4:**
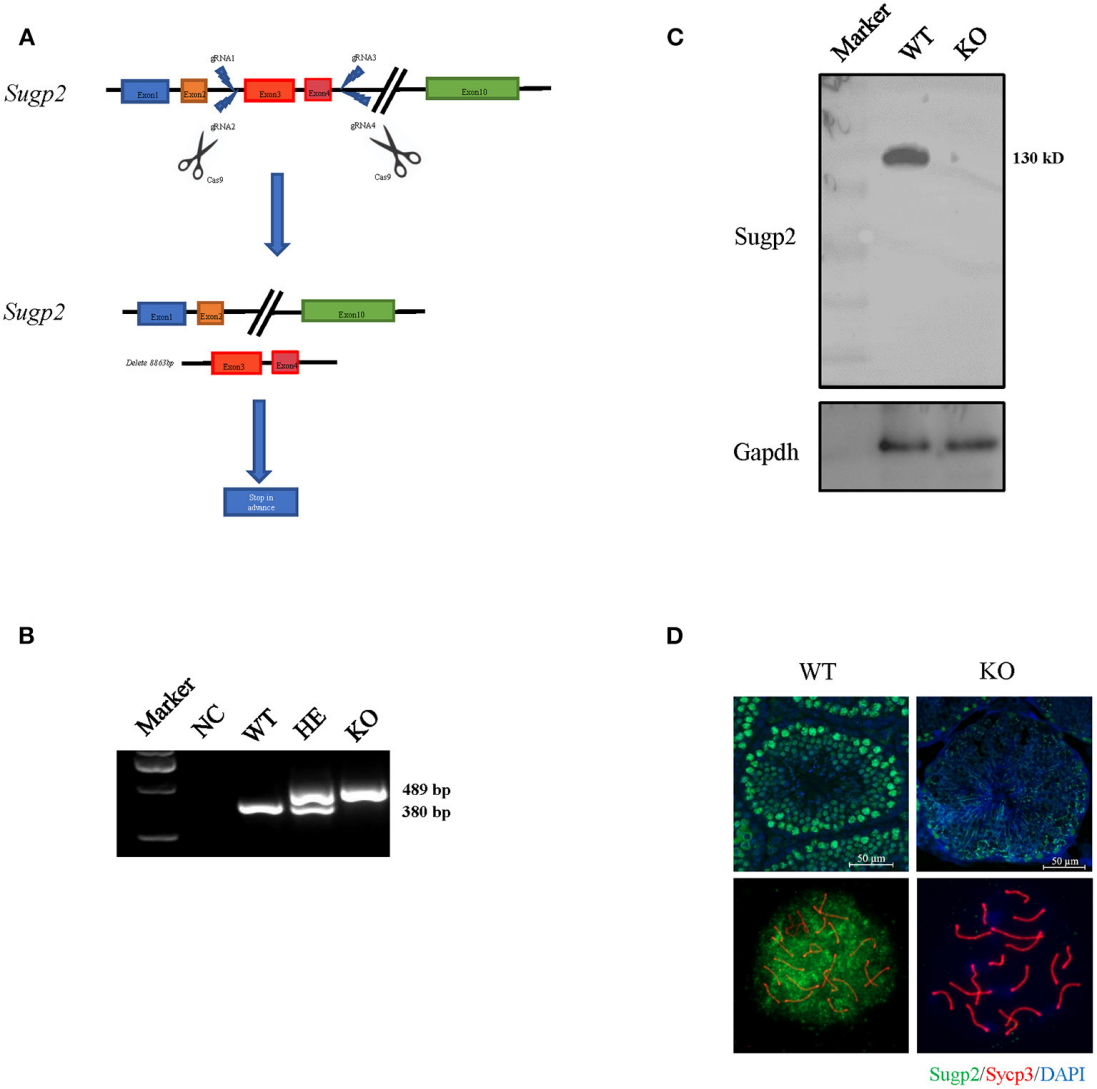
Generation of Sugp2 knockout mouse. **(A)** Knockout strategy of Sugp2. Exons 3 and 4 were deleted by the CRISPR/Cas9 genome editing system. **(B)** Mouse genotype identification by mouse tail PCR. WT, wild type; HE, heterozygote; KO, knockout. Western blot **(C)** and immunofluorescence staining **(D)** of Sugp2 in adult WT and Sugp2 KO testes. *n*, number of samples per group.

The previous expression and localization results indicated that Sugp2 may be involved in mouse spermatogenesis. To study the biological function of Sugp2, we generated Sugp2-deficient mouse (Sugp2^−/−^) with the CRISPR/Cas9 genome editing system, which deleted an 8.863-kb genomic DNA fragment containing exon 3 and exon 4. The deletion induced the premature termination of translation with production of incomplete protein (Figure 4A). The genotyping of Sugp2 could be performed by mouse tail PCR (Figure 4B). Western blotting and immunofluorescence analysis confirmed the absence of the Sugp2 protein in Sugp2^−/−^ testes, indicating the successful knockout of Sugp2 (Figures 4C,D).

**Figure 5:**
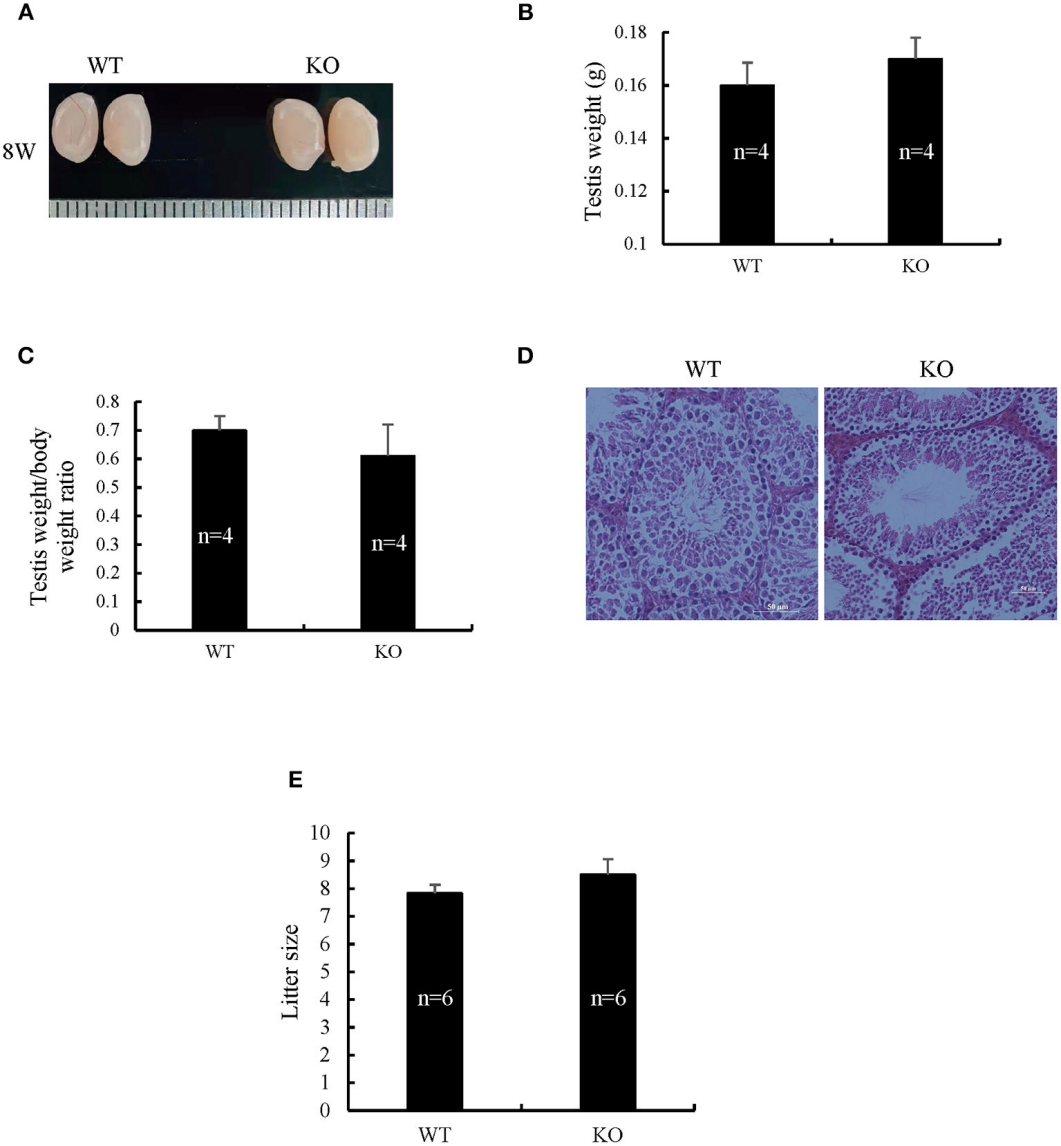
Morphological analysis of Sugp2 knockout mouse. **(A)** Size of adult testes from wild-type (WT) and Sugp2 KO mice. Testis weight **(B)** and the ratio of testis to body **(C)** from WT and Sugp2 KO mice. Error bars represent standard error. **(D)** Hematoxylin and eosin (H&E) staining of adult testes in WT and Sugp2 KO mice. Scale bar = 50 μm. **(E)** Comparison of litter size from wild-type and Sugp2 knockout mice.

Spermatogenetic problems typically resulted in sterility or reduced fertility, reduced testis weight, and/or seminiferous tubule abnormalities. The disruption of Sugp2 had a little impact on testis size (Figure 5A). Meanwhile, the testis weight and testis/body weight ratio of adult Sugp2^−/−^ males were indistinguishable from their wild-type (WT) littermates (Figures 5B,C). A histological analysis showed that spermatogenesis in Sugp2^−/−^ males was not grossly impaired compared to WT males (Figure 5D). To address whether Sugp2 affected male fertility, Sugp2 knockout males were placed in cages with WT females for mating, and the litter size was recorded. The sugp2 knockout mice are fertile, and there was no significant difference in the liter size (Figure 5E).

### Sugp2^–/–^ Males Do Not Show Gross Defects in Meiotic Prophase Progression

**Figure 6:**
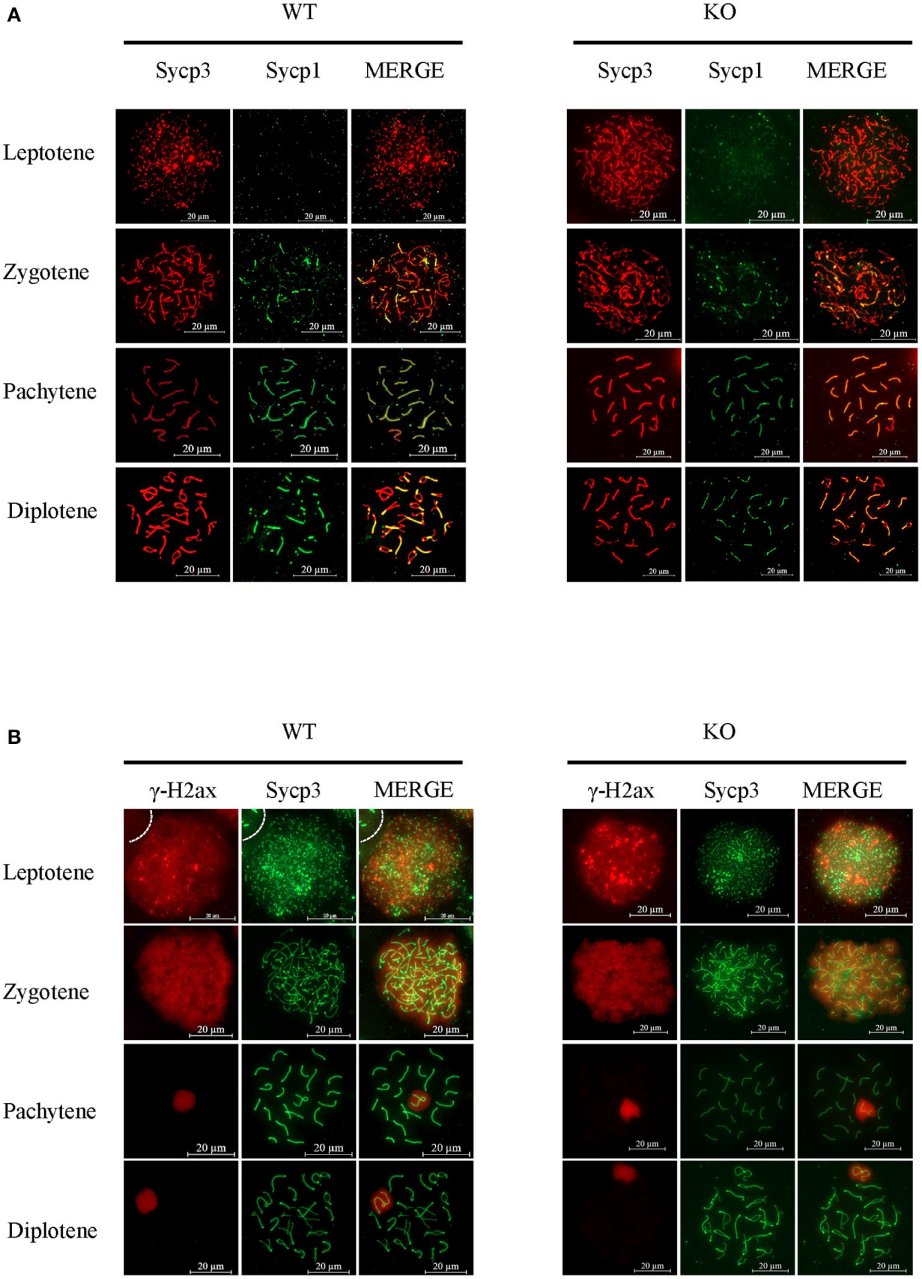
Function of Sugp2 in chromosome synapsis and double-strand break repair in spermatocytes. **(A)** Chromosome spread from wild-type control and Sugp2 knockout testes was labeled for synaptonemal complex proteins (Sycp1 and Sycp3). **(B)** Distribution of γH2ax in wild-type and Sugp2 KO spermatocytes. Scale bar = 20 μm.

To uncover more subtle meiotic changes in Sugp2-deficient spermatocytes, we immune-stained meiotic chromosome spreads. In meiotic prophase, germ cells can be classified into four different cytological stages—leptonema, zygonema, pachynema, and diplonema—based on the kinetic changes of the SC which consisted of central protein (Sycp1) with axial/lateral element (Sycp3). In Sugp2^−/−^ spermatocytes, chromosome synapsis can process normally and SC can form and disassemble normally with no obvious defects (Figure 6A).

**Figure 7:**
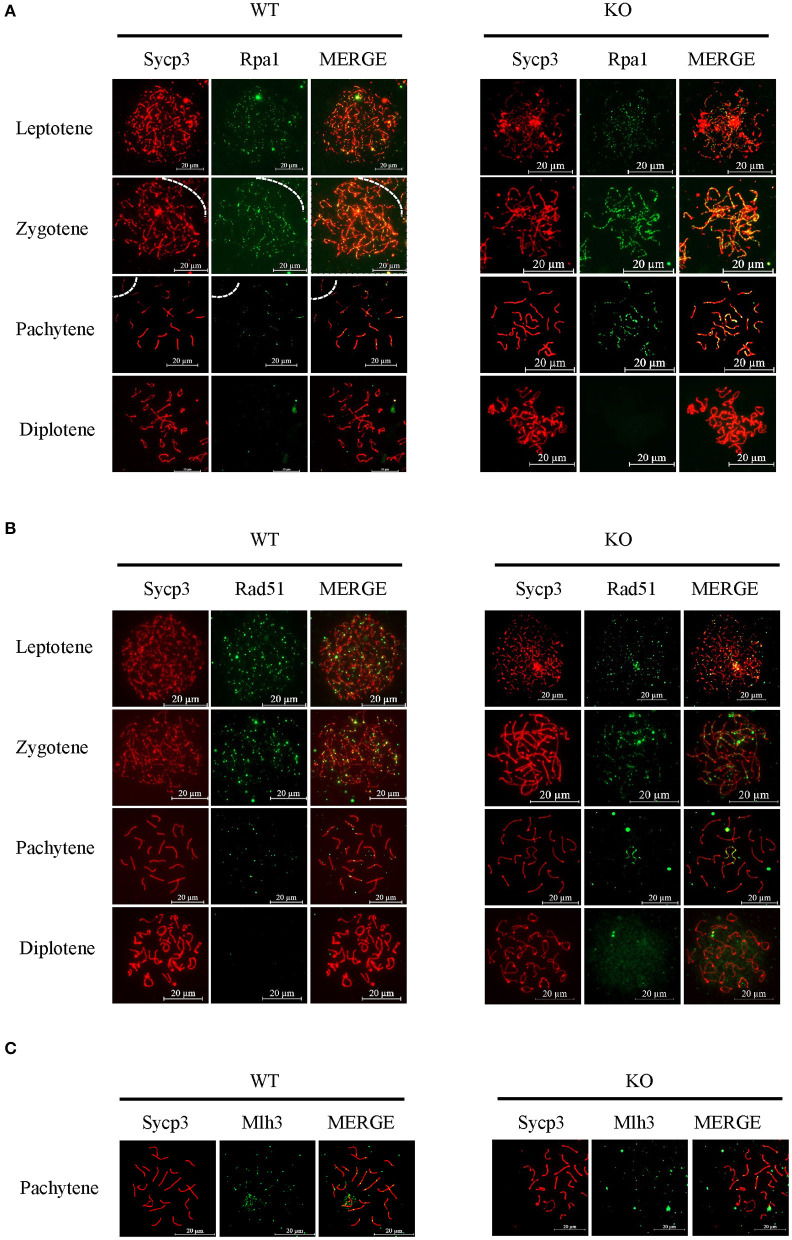
Sugp2 is not essential for meiotic recombination. Immunolabeling for the synaptonemal complex component Sycp3 and recombination protein Rpa1 **(A)** and Rad51 **(B)**. **(C)** The crossover formation was analyzed by staining Mlh3. Scale bar = 20 μm.

Next, we used typical markers to evaluate the formation and repair of meiotic DSBs. In WT males, γH2ax appears during leptonema and then disappears from the autosomes as DSBs are repaired, leaving its signal exclusively in the XY body during pachynema and diplonema. The appearance and disappearance of the γH2ax signal was normal in Sugp2^−/−^ males, suggesting that DSB formation and repair were completed normally in the absence of Sugp2 (Figure 6B). We also stained the Rpa1 and Rad51 involved in meiotic recombination, whose foci are maximal at early- to mid-zygonema and then decrease as DSBs are repaired *via* homologous recombination. Rpa1 and Rad51 in Sugp2^−/−^ spermatocytes displayed an overall similar progression with that of WT spermatocytes (Figures 7A,B). At the mid-late pachytene stage, the Mlh3 foci become apparent in spermatocytes, marking sites of crossover. Compared to their WT controls, the Sugp2^−/−^ males showed little difference in Mlh3 foci (Figure 7C). These results demonstrated that Sugp2 was dispensable for the initiation and completion of meiotic prophase progression.

### Sugp2 Regulates Pre-mRNA Splicing During Spermatogenesis

**Figure 8:**
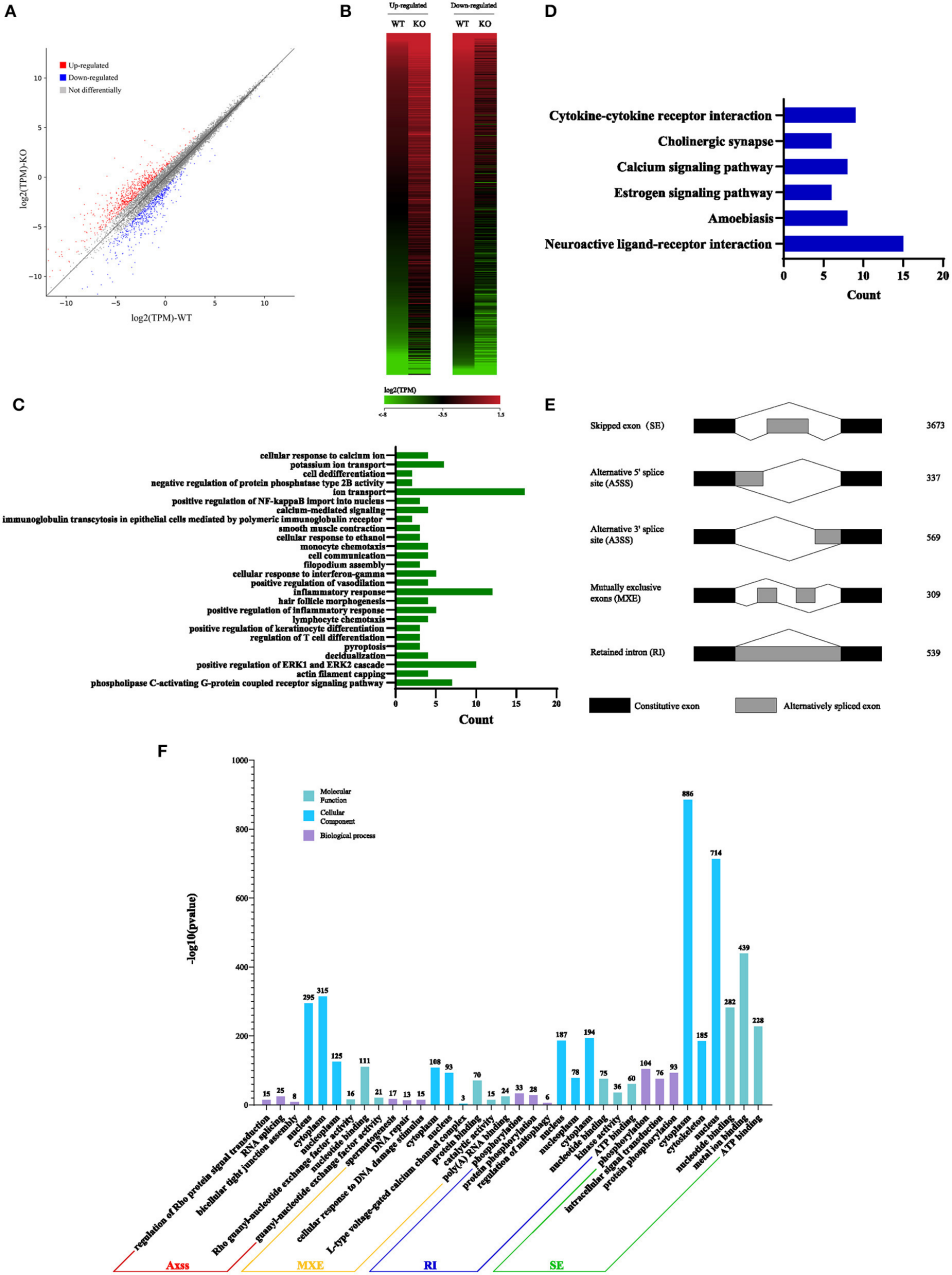
Sugp2 is involved in RNA splicing during spermatogenesis. Volcano plot **(A)** and heat map **(B)** of differentially expressed transcripts in Sugp2 KO testis compared with WT control. Green dots represent significantly downregulated transcripts, red dots represent significantly upregulated transcripts (|log2FC| >1), and gray dots represent unchanged transcripts. Gene Ontology (GO) **(C)** and Kyoto Encyclopedia of Genes and Genomes **(D)** functional analysis of differentially expressed genes. **(E)** Five significantly affected RNA splicing events in Sugp2 KO spermatogenic cells from adult mice. **(F)** The three most significantly (*P* < 0.05) enriched GO terms in biological process, molecular function, and cellular component branches from each AS group are presented.

Considering that Sugp2 is a potential member of the SR-related family of pre-mRNA processing factors, we isolated mRNA from adult WT control and Sugp2^−/−^ testes and then performed whole-transcriptome RNA sequencing. A total of 66,763,192 clean reads were used for downstream bioinformatics analysis for the control and Sugp2^−/−^ testes, respectively. With a common parameter (|log_2_FC| >1), the total transcripts showed about 1,331 differential expressions in Sugp2-deficient testis, in which 729 genes were upregulated and 602 genes downregulated (Figures 8A,B). Then, we performed Gene Ontology (GO) and Kyoto Encyclopedia of Genes and Genomes (KEGG) analysis to further analyze the effect of Sugp2 knockout on cell function. The functional analysis results indicated that Sugp2 may participate in ion metabolism and neuroactive signal pathway, but all functions were only with a few changed genes (Figures 8C,D). Since the SURP and G-patch domain contained in Sugp2 are mainly involved in transcript alternative splicing, we analyzed the influence of Sugp2 deletion on AS. Five different AS types were compared between WT control and Sugp2^−/−^ testes. Compared with control, 5,427 AS events were significantly affected (*P*-value < 0.01) in Sugp2^−/−^ testes. Among those affected AS events, the majority (3,673/5,427) of the changed splicing events were skipped exon (SE), which indicated that Sugp2 was involved in mRNA splicing during mouse spermatogenesis (Figure 8E). Then, we performed GO analysis about the affected AS from five different groups to further uncover the function of alternative splicing genes. The top three most significantly (*P* < 0.05) enriched GO terms were displayed. In the SE group, Sugp2 mainly regulated the expression of genes on metal ion, ATP, and nucleotide binding, implying its function on metal ion, and energy metabolism (Figure 8F). All these data indicated that Sugp2 participated in mRNA alternative splicing and may be involved in cell ion and energy metabolism during mouse spermatogenesis.

## Discussion

Mammalian spermatogenesis is a complex process involving a variety of cell types and developmental stages in a highly collaborative order accompanied with complicated structural changes in chromatin involved in complex regulation mechanisms (13, 22). Therefore, in the present study, we developed a multiple control group proteomics strategy to systematically identify different spermatogenetic chromatin-associated proteins in different cell or meiotic stages. Sugp2, in the data, drew our attention since its expression arose along with meiotic progression, but with a few signals in Spo11^−/−^ testis in which there were neither meiotic recombination events nor post-zygotene spermatocytes.

Sugp2, also known as SFRS14, contains SURP1/2 domains which are mostly presented in alternative splicing regulators as well as one G-patch domain, a region of six highly conserved glycine residues commonly found in RNA-processing proteins (23, 24). Highly differentiated tissues, such as testis and brain, have more post-transcription regulation than any other tissues (25). All of these prompted us to perform a functional study of Sugp2 in spermatogenesis.

Our results firstly evidenced that Sugp2 had two transcripts encoding some proteins in the testis, and it had been demonstrated that its transcription isoforms could be regulated during spermatogenesis (26). This also indicated that there was a complicated regulation mechanism on Sugp2 expression. Sugp2 protein was primarily expressed in mouse testis and enriched in the nucleus of male germ cells. Interestingly, Sugp2 was diffused in the autosome area but was excluded from the region of the XY body from the pachynema to diplonema. This localization pattern was similar with RNA poly II which would also be absent from the XY body because of meiotic sex chromosome inactivation, resulting in the inactivation of the asynapsed XY chromosome regions (27, 28). The chromatin localization of Sugp2 may also support its regulation in gene expression during spermatogenesis.

The knockout of sugp2 triggered some changes in the expression level of some transcripts compared with the WT control. After GO and KEGG analysis, we found that each of the most affected group only had few changed genes, which could not provide more information about Sugp2-regulated molecular functions. Then, we conducted AS analysis since the domains SURP1/2 and G-patch always participated in the AS process. Alternative splicing, by which pre-mRNA molecules can be spliced in different ways to generate multiple mRNA isoforms from a single gene, is the main mechanism of transcriptome and proteome expansion that can explain the phenotypic complexity of organisms and tissues (29–31). In the five main AS events, Sugp2 mainly regulated the skip of exons which would probably generate abnormal exon-skipped transcripts with a premature termination codon. The abnormal alternative splicing in SE was closely associated with male infertility (32, 33). The GO analysis based on AS data indicated that Sugp2 may participate in metal ion and energy metabolism. Combined with the results of KEGG, Sugp2 may be involved in calcium transport and signal transduction, which play an essential role in male germ cell development and function (34, 35).

Unexpectedly, Sugp2-deficient males were fully fertile and exhibited no gross spermatogenetic abnormalities. The deficiency of Sugp2 had no obvious effect on meiotic synapsis, DSB repair and meiotic recombination, and male fertility. Sugp2 knockout significantly altered the expression profiles compared with WT controls. Changes in the transcription level or the alternative splicing variants of these altered genes could not probably cause gross harm to mouse spermatogenesis. What is more, through analysis of the proteomics screen data, we found that Sugp1, which shares a high homology with Sugp2 and also has SURP1/2 and G patch domains, also showed a high signal during spermatogenesis, indicating that it may also have functions in the testis (36). Whether Sugp2 is a redundant gene for Sugp1 can be further verified by double-knockout experiments.

In summary, our results showed that Sugp2 was a chromatin-associated protein and enriched in the nucleus of male germ cells. Although Sugp2 was dispensable for male fertility, it indeed regulated many AS events during spermatogenesis.

## Data Availability Statement

The datasets presented in this study can be found in online repositories. The names of the repository/repositories and accession number(s) can be found below: https://www.ncbi.nlm.nih.gov/, PRJNA752112.

## Ethics Statement

The animal study was reviewed and approved by the animal ethical guidelines of the Institutional Care and Wuhan University.

## Author Contributions

CL, JZ, JL, and YW designed and conceived the research and performed most of the experiments. JZ, JL, and CL purified all chromatin-associated proteins and identified Sugp2. JL, PW, and CL made the antibodies. YW managed and bred the animals used in the research and analyzed the phenotype. ZY performed the bioinformatics analysis. PC, MZ, YX, TJ, and ZD analyzed the data and phenotypes. CL and JZ wrote the manuscript. ML participated in the manuscript discussion. All the authors participated in the manuscript preparation and approved the final manuscript.

## Funding

This research was supported by National Key Research and Development Program of 363 China (Grant No. 2018YFC1003400), National Natural Science Foundation of China (Grant No. 31771588), Strategic Collaborative Research Program of the Ferring Institute of Reproductive Medicine (Grant No. FIRMC200509), and Chinese Academy of Sciences (Grant No. FIRMC200509 to ML).

## Conflict of Interest

The authors declare that the research was conducted in the absence of any commercial or financial relationships that could be construed as a potential conflict of interest.

## Publisher's Note

All claims expressed in this article are solely those of the authors and do not necessarily represent those of their affiliated organizations, or those of the publisher, the editors and the reviewers. Any product that may be evaluated in this article, or claim that may be made by its manufacturer, is not guaranteed or endorsed by the publisher.
